# Use of a Point-of-Care Progesterone Assay to Predict Onset of Parturition in the Bitch

**DOI:** 10.3389/fvets.2022.914659

**Published:** 2022-06-23

**Authors:** Johan O. Nöthling, Carolynne J. Joonè, Evan Hegarty, Elizabeth K. Schooley, Kurt G. M. De Cramer

**Affiliations:** ^1^Department of Production Animal Studies, Faculty of Veterinary Science, University of Pretoria, Onderstepoort, Pretoria, South Africa; ^2^College of Public Health, Medical and Veterinary Sciences, Division of Tropical Health and Medicine, James Cook University, Townsville, QLD, Australia; ^3^IDEXX Laboratories Inc., Westbrook, ME, United States

**Keywords:** dog, whelp, chemiluminescent immunoassay, vaginoscopy, Catalyst, P4, in-house analyzer, IDEXX

## Abstract

An assay of circulating progesterone (P4) is commonly used to estimate progress through late gestation in the bitch. Point-of-care assays provide rapid results, a major advantage over laboratory-based assays. This study aims to compare P4 levels determined by the Catalyst® Progesterone point-of-care assay with those determined by chemiluminescent immunoassay (CLIA) and to identify the expected distribution of Catalyst P4 levels at time intervals 3 days prior to the onset of parturition in pregnant bitches. Twenty-eight pregnant bitches carrying two or more fetuses were admitted to a specialist veterinary reproduction hospital 53 days after the onset of cytological diestrus or, when that date was not known, 57 days after the last mating. Vaginal speculum examinations were performed every 6 h until the onset of cervical dilatation (TCD). Serum samples were collected twice daily (08h00 and 18h00) until TCD. For most samples, fresh serum was assayed for P4 immediately using the Catalyst assay (CatP4), then frozen until assayed by CLIA (IMMULITE 2000; ImmP4). However, for some samples, CatP4 was not analyzed prior to freezing. For these data points (*n* = 33), CatP4 for fresh serum was estimated from CatP4 assayed on frozen-thawed serum, based on a comparison between CatP4 on fresh vs. frozen-thawed sera. In comparison to ImmP4, CatP4 levels up to and including 7 nmol/L appear to have a constant bias of −1.69 nmol/L (limits of agreement −4.91 to 1.52), while levels >7 nmol/L appear to have a proportional bias of −17.9% (limits of agreement −68.6% to 32.7%). Bootstrapped percentiles of CatP4 results spanned 0.4–9 nmol/L within 12 h of TCD, 0.9–11 nmol/L 12–24 h from TCD, and 2.2–13.5 nmol/L 24–36 h from TCD. A CatP4 >9 nmol/L indicates a bitch that is unlikely to reach TCD within 12 h. Bitches with CatP4s below 3.5 nmol/L are likely to reach TCD within 36 h and bitches with a CatP4 below 2.2 nmol/L are likely to reach TCD within 24 h. In conclusion, the Catalyst Progesterone assay provides rapid assessment of circulating P4 in the bitch, with clinical application in the monitoring of late term pregnant bitches.

## Introduction

To maximize neonatal survival, it is vital that canine fetuses are fully mature at delivery. The bitch has a relatively short gestation length of approximately 63 days from ovulation (1), or approximately 57 days from the onset of dioestrus (2), with fetal lung maturation thought to be reached in the final few days (3). This has important implications for the management of late-term pregnant bitches, particularly those at high risk of requiring Cesarean section or where elective Cesarean section may significantly increase fetal survival rates (4).

Recent research suggests that Cesarean section in the bitch may be performed safely up to 52 h prior to the onset of stage one of spontaneous parturition (cervical dilatation) (5). However, to apply this knowledge, a clinician must be able to accurately and precisely estimate when spontaneous parturition would likely begin. Clinical data obtained as part of the monitoring of a bitch during breeding predicts her subsequent whelping date with relative accuracy (6). However, many late-term bitches present with only mating dates available. The use of mating dates to estimate when a bitch is due to whelp is unreliable, given that gestation length from the first mating to parturition varies from 58 to 72 days (7).

In the pregnant bitch, circulating progesterone (P4) levels decline abruptly prior to parturition (8–12). Serum or plasma P4 levels are therefore frequently monitored in an attempt to estimate the time to onset of parturition. Traditionally, Cesarean section has been considered safe for the dam and litter when P4 levels fall below 6.4 nmol/L (2 ng/ml) (13), although few studies have sought to verify this assumption. Rota et al. (14) found that all bitches with a P4 below 10.8 nmol/L (3.4 ng/ml) performed on a chemiluminescent immunoassay (CLIA; IMMULITE 2000 by Siemens Medical Solutions Diagnostics) whelped within 1 day, however, more than half of the bitches that went on to whelp within 1 day had a P4 above this level. Recently, KD and JN (12) identified four cut-off values for plasma P4 levels measured on the Coat-A-Count® radioimmunoassay that are helpful in predicting the time to onset of parturition in bitches.

Historically, the gold standard for determining plasma or serum P4 levels in the bitch was the Coat-A-Count ^125^I RIA by Siemens. This test was discontinued in 2014 (15). Since different assays employ different technologies, there can be inherent differences in the analyte levels reported by each assay. Therefore, there is a need to evaluate how results from one assay compare to results from a different type of assay. The IMMULITE (CLIA) analyzer by Siemens has been validated for the measurement of P4 in dogs and is now widely used in research and clinical practice (16–19). Like RIA, CLIA is generally performed by commercial diagnostic laboratories and therefore has a relatively long turnaround time, exacerbated by sample transport times. In contrast, Catalyst® Progesterone is a point-of-care assay that provides results within 12 min, potentially increasing the diagnostic value of P4 monitoring in late term bitches. To date, one study comparing Catalyst Progesterone to CLIA has been published; this work was limited to a method comparison of frozen clinical samples without evaluating its use in a clinical setting (20).

The aims of this study were to (1) compare P4 levels determined by the Catalyst Progesterone assay with those determined by CLIA, (2) determine the distribution of expected Catalyst P4 levels at various timepoints prior to the onset of parturition in late pregnant bitches, and (3) use Catalyst P4 levels to define cut-offs by which to predict the onset of parturition in late pregnant bitches.

## Materials and Methods

This study was approved by the Animal Ethics Committee of the University of Pretoria (project V106-18).

### Animals and Sampling

Twenty-eight bitches (22 Boerboels, three English bulldogs, two American bullies, and one Labrador retriever) carrying litters of two or more that were admitted to a specialist veterinary reproduction hospital in preparation for elective Cesarean section during the second half of 2019 were included in this study. Bitches were admitted 53 days after the onset of cytological diestrus (2) when the day was known or 57 days after the last mating. Bitches stayed in indoor cages and went for an outside walk 2 times daily. They were fed commercial dry pellets and had access to clean water *ad lib*.

Vaginoscopy was performed as previously described (5, 12) to identify the onset of stage one of parturition in each bitch. Bitches underwent vaginoscopy at admission and then every 6 h until dilatation of the uterine cervix was visualized, or more frequently if it was suspected that cervical dilatation was imminent. The time of cervical dilatation (TCD) was defined as the time of vaginoscopy at which any degree of cervical dilatation was first observed.

Blood collection occurred within 30 min of 08h00 and again within 30 min of 18h00 every day following hospital admission, with a final sample collected at TCD. Samples collected at regular bleeding times before TCD were classified by the time, relative to TCD, at which each serum sample was obtained as follows: Time 1 accounted for sera obtained at the last regular bleeding time before TCD, Time 2 for sera obtained at the penultimate regular bleeding time before TCD, and so on, up to Time 5. Although the intervals between regular bleedings were approximately 10 h or 14 h, we assumed that the intervals were all 12 h for statistical purposes. The actual intervals and the assumed intervals between each regular bleeding and TCD are shown in Table 1. Only sera collected up to Time 5 (approximately 60 h prior to TCD) were included in the study.

**Table 1:** Intervals between regular bleeding times and the time of cervical dilatation (TCD) in h.

	**TCD occurred after 08h00 and no later than 18h00 that evening**			**TCD occurred after 18h00 and no later than 08h00 the next morning**			**Assumed number of h before TCD that were used in the study**	
**Regular bleeding no**.	**Time of day**	**Min. h to TCD**	**Max. h to TCD**	**Time of day**	**Min. h to TCD**	**Max. h to TCD**	**Min. h to TCD**	**Max. h to TCD**
1 (last)	08h00	0	10	18h00	0	14	0	12
2 (2nd last)	18h00	14	24	08h00	10	24	12	24
3 (3rd last)	08h00	24	34	18h00	24	38	24	36
4 (4th last)	18h00	38	48	08h00	34	48	36	48
5 (5th last)	08h00	48	58	18h00	48	62	48	60

Blood was collected into plain vials with clot activator (BD Vacutainer®, BD, Plymouth, UK) and centrifuged once clotting had occurred (at room temperature). Apart from an aliquot destined for the Catalyst Progesterone assay, serum was transferred into plastic cryovials (Catalog number 750273, PlastPro Scientific, Edenvale, South Africa), uniquely labeled, and frozen at −20 °C until analyzed.

A Cesarean section was performed on each bitch at TCD, and the number of puppies, the number of stillborn puppies, and the number of puppies that died before they were two hours old were recorded.

### Measuring P4

#### With the Catalyst Assay

An aliquot of fresh serum was used to determine CatP4 using the Catalyst assay (Catalyst® Progesterone, IDEXX Laboratories Inc., Westbrook, Maine, USA) run on a Catalyst Dx Chemistry Analyzer (IDEXX Laboratories, Inc.), according to the manufacturer's instructions.

In order to evaluate the coefficient of variation (CV) for this assay, 32 serum samples representing a range of CatP4 values were tested twice in quick succession and by the same operator. To guarantee a range of progesterone levels and sufficient volume for repeated testing, some samples included for CV determination came from periparturient bitches that were not included in the rest of the study.

**Figure 1:**
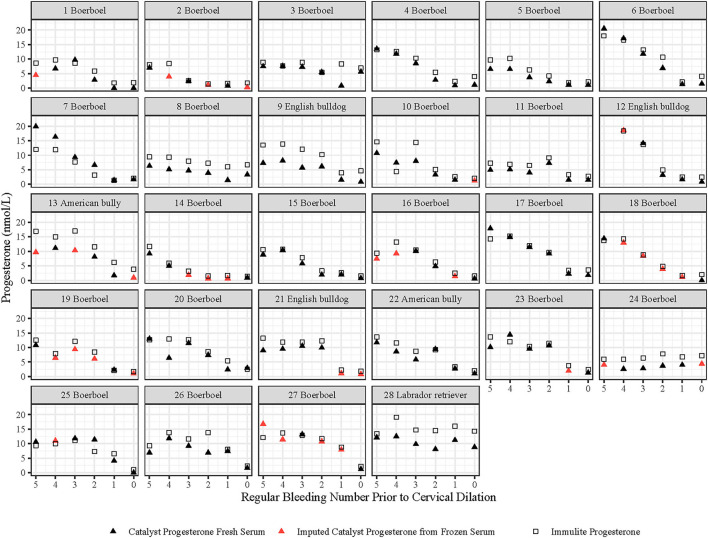
Serum progesterone levels measured with the Catalyst and IMMULITE assays in 28 bitches before and at the first detected time of cervical dilatation (TCD, shown as time zero). Time 1–5 represent the regularly scheduled bleeding times at 0800h and 1800h that occurred before TCD. Time 1 could occur 0–14 h before time zero.

Fresh serum was analyzed for CatP4 on 134 of the 167 samples from the 28 bitches in the study (Figure 1). For the remaining 33 samples, only frozen serum was available. To estimate CatP4 (fresh serum) from CatP4 analyzed on frozen-thawed serum for these samples, we determined CatP4 in duplicate on 16 fresh serum samples and again on the same samples after they had been frozen and thawed, and compared the results. Some of the 16 serum samples used in this comparison were not from the 28 bitches used in this study but from other preparturient bitches. Based on this comparison, we calculated CatP4 (fresh serum) for the remaining 33 datapoints based on duplicate CatP4s assayed on frozen-thawed serum. As shown in Figure 1, using these 33 CatP4s together with those done on fresh serum resulted in CatP4 being known twice daily from 60 h prior to TCD until TCD in all 28 bitches, except bitch 12 for which no serum was collected at Time 5.

#### With the IMMULITE Assay

Progesterone levels were determined on frozen-thawed serum using the IMMULITE 2000 Progesterone assay (Siemens Healthcare Diagnostics Products Ltd., Glyn Rhonwy, UK). We used the same kit for all assays, with one run done on each of 3 days. Each serum sample was assayed in duplicate within a single run. We refer to the mean level of P4 for each pair of duplicates as ImmP4. The intra-assay CV was calculated using the P4 of each pair of replicates. To calculate the interassay CV, 12 serum samples representing a range of ImmP4 values obtained in the first run were assayed again in the second and third runs. Some of the serum samples used to determine the CVs for this assay were not from the 28 bitches used in this study but from other preparturient bitches.

### Data Analysis

#### Comparing CatP4 and ImmP4

The agreement between CatP4s measured on fresh sera and ImmP4s measured on corresponding frozen-thawed sera was determined by means of Bland-Altman agreement analysis (21) and the association between them by means of Passing–Bablok regression (22).

#### Estimating CatP4 of Fresh Serum Based on CatP4 of Frozen Serum

A scatterplot of the mean CatP4 of the 16 duplicate fresh and frozen-thawed sera pairs was visually evaluated to determine if there was a linear relationship. A strong linear relationship was apparent for the 15 samples with CatP4s (fresh serum) of 16.2 nmol/L or below. The remaining CatP4 (23.7 nmol/L on fresh serum) deviated substantially from the straight line. Excluding this outlier, we used linear regression to determine the association between the mean CatP4 of fresh and frozen-thawed serum samples. We used the inverse of the regression equation so obtained to estimate CatP4 (fresh serum) for the 33 serum samples for which only CatP4 (frozen serum) was available.

#### CatP4 Values in the Preparturient Period

Sample results were grouped by timepoint before TCD for analysis. A range of expected values for each timepoint was calculated following the Clinical and Laboratory Standards Institute (CLSI) recommended Reference Interval analysis for small samples of non-normal data (23). Reference Interval analysis was considered appropriate given that this analysis was intended to identify expected CatP4 levels for different timepoints. An expected 95% of observed progesterone values should fall within this range for the relevant time point. Limits of the expected range of P4 values for each time period were estimated from 100,000 bootstrapped random samplings from the dataset of the same size as the original dataset sampled with replacement. From each random sampling, the 2.5- and 97.5-percentiles were taken, resulting in 100,000 values for each of these percentiles. The medians of these 100,000 percentile values were reported as the lower (2.5-percentile) and upper (97.5-percentile) bounds of the expected range of P4 values. The 95% confidence intervals for these estimates were derived from the limits of the central 95% of these values. Analyses were performed using STATA version 14 (StataCorp, College Station, Texas, USA) and R version 4.1.0 (24).

## Results

All bitches made an uneventful recovery after Cesarean section. In total, 222 puppies were delivered. Litter sizes ranged from 3 to 15 puppies per litter (median 7.5, first quartile 4.5 and third quartile 11 puppies per litter). One bitch had two stillborn puppies and two others one each. Of the 219 puppies that were born alive, all but two were alive 2 h after the surgery.

### Intra- and Interassay Coefficients of Variation

The CVs for CatP4 measured by the Catalyst assay are shown in Table 2 and the intra- and inter-assay CVs for ImmP4 measured by IMMULITE are shown in Table 3.

**Table 2:** Coefficient of variation for the Catalyst Progesterone assay based on two assays per fresh serum sample, in quick succession and by the same operator.

**Limits in P4 levels (nmol/L) of the buckets used**	**CV (%)**	***n*** ^a^
0.7–6	7.8	11
7–12	13.4	12
14–27	12.0	9

a *Some of the serum samples used to determine the CV for this assay were not from the 28 bitches used in this study but from other preparturient bitches*.

**Table 3:** Intra- and inter-assay coefficients of variation for the IMMULITE progesterone assay.

**Limits in P4 levels (nmol/L) of the buckets used**	**CV (%)**	***n*** ^a^
Intra-assay CV		
0.8–6	6.7	93
6–10	5.2	59
10–13	5.3	42
13–24	3.9	45
Inter-assay CV		
1.5–6	9.1^b^	3
10–14	2.9	5
17–22	4.8	4

a *Some of the serum samples used to determine the CV for this assay were not from the 28 bitches used in this study but from other preparturient bitches*.

b *This value was strongly affected by the CV for the 1.5 nmol/L sample, which was an outlier at 19.7%, compared to the CVs of the other two serum samples, which were 3.3 and 4.4%*.

### Assessment of CatP4

#### The Agreement Between CatP4 Done on Fresh Serum and ImmP4 on Frozen Serum

Visual appraisal of Figure 1 reveals that, although CatP4 done on fresh serum is often very close to ImmP4, some differences exist between the values.

**Figure 2:**
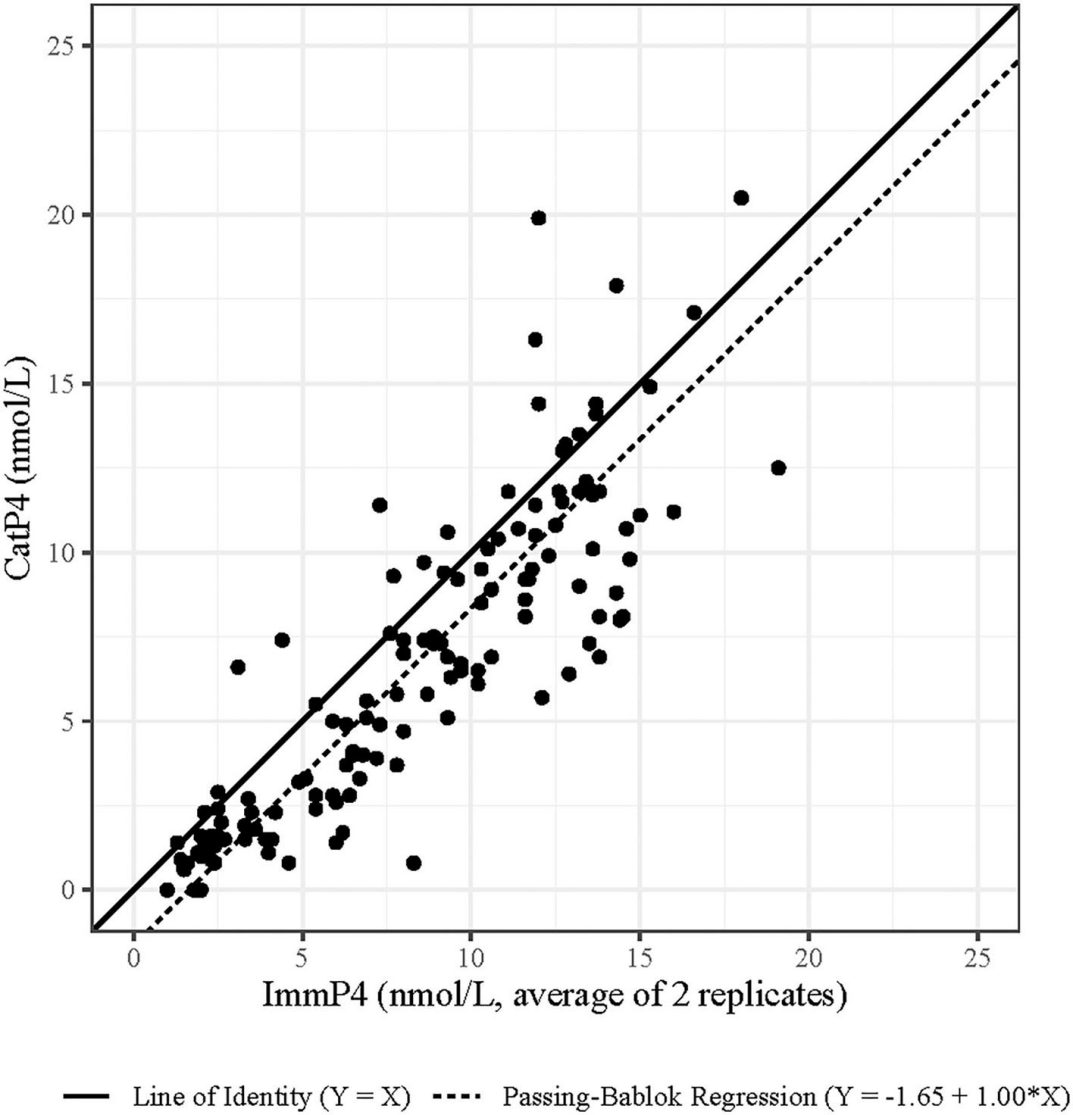
Passing–Bablok regression of 134 progesterone levels measured with the Catalyst and IMMULITE assays.

**Figure 3:**
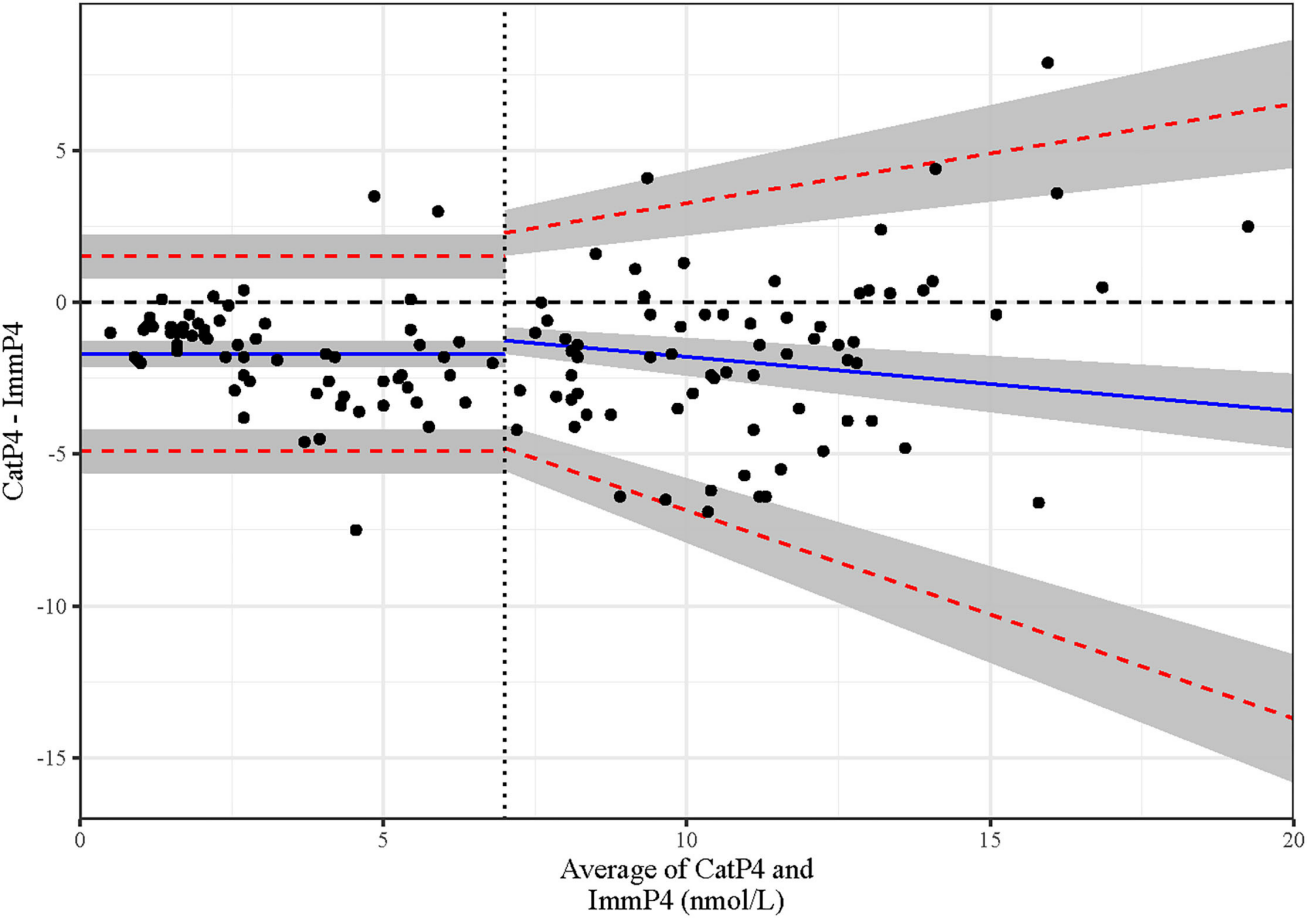
Bland-Altman plot showing agreement between 134 progesterone levels measured with the point-of-care Catalyst assay and those measured with chemiluminescent immunoassay (IMMULITE).

The results of the Passing–Bablok regression (Figure 2) showed an intercept of −1.65 (95% CI: −2.08 to −0.88) and a slope of 1.00 (95% CI 0.90 to 1.07). This suggests that CatP4 had a constant negative bias from the value of ImmP4, but that this bias did not change over the range of P4 values in the dataset. Figure 2 shows, however, that the variation in bias over the range of P4 values was not constant, and that a proportional bias exists for at least some portion of the range of values. For this reason, the bias was categorized in two buckets of P4 values to better categorize the bias across the whole range of values as recommended by the CLSI guidelines for method comparison (25). A Bland Altman plot was used to visually identify the P4 level at which the variance in bias changed (Figure 3). CatP4 levels up to and containing 7 nmol/L have a bias best described by a constant shift of −1.69 nmol/L (limits of agreement (LOA): −4.91 to 1.52) from the comparison (averaged P4 between the two methods), while CatP4 levels >7 nmol/L have a bias best described as a proportional −17.9% (LOA: −68.6 to 32.7%) to the value of the comparison.

#### The Effect of Using Frozen Serum to Determine CatP4

**Figure 4:**
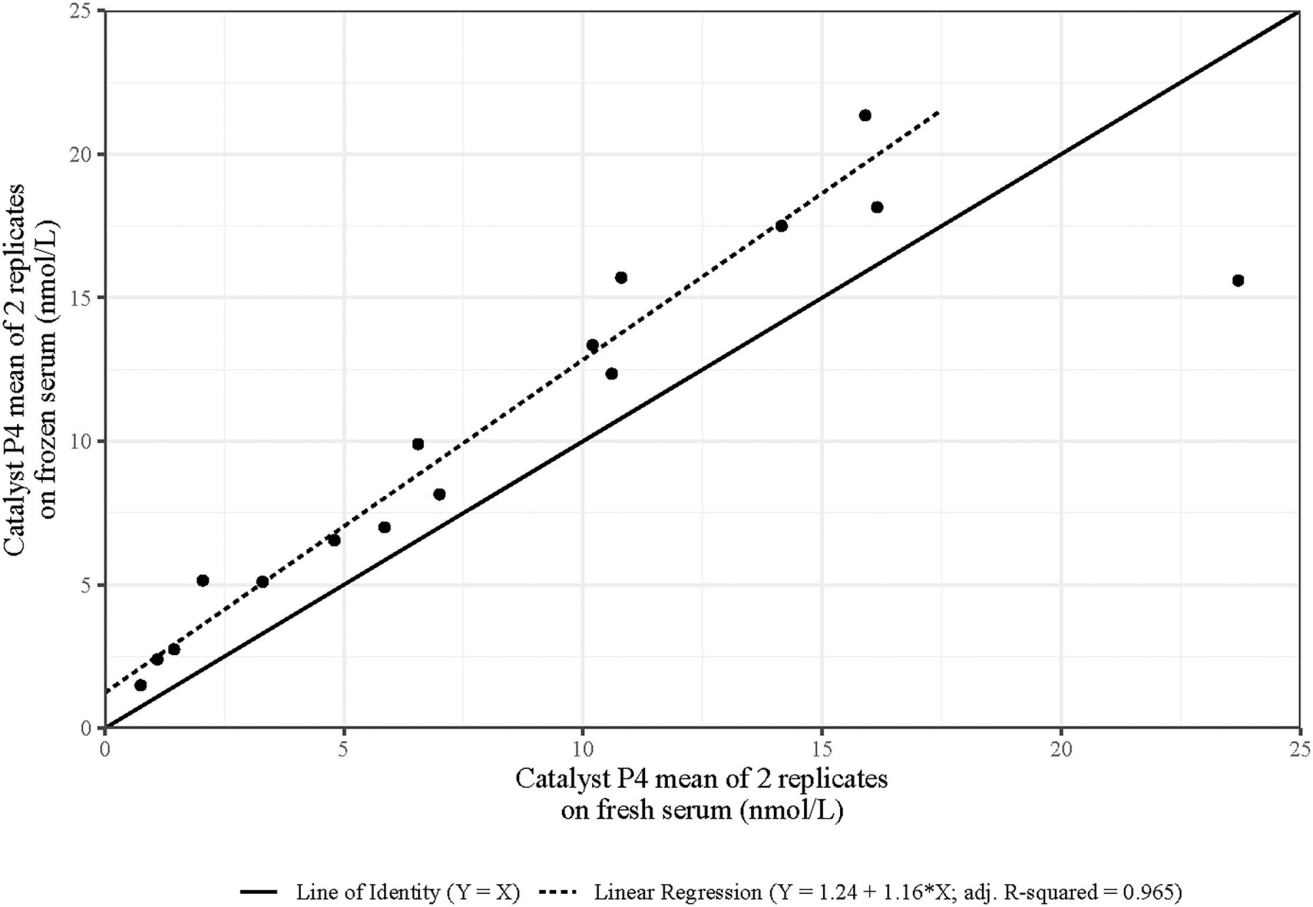
Progesterone levels were determined using the Catalyst point-of-care assay (CatP4) paired with fresh and frozen-thawed sera. Duplicate CatP4 was determined for both the fresh and frozen-thawed aliquots. Linear regression was performed after the exclusion of the outlier.

There was a strong linear relationship between CatP4 (fresh serum) and CatP4 in the same serum after it had been frozen for levels of CatP4 (fresh serum) of 16.2 nmol/L or below (Figure 4). CatP4 (frozen serum) increases as CatP4 (fresh serum) increases (*p*-value for the slope < 0.001, *n* = 15). CatP4 in frozen serum is estimated as 1.23 + 1.16 × CatP4 in fresh serum. At the highest value of CatP4 (fresh serum) of 23.7 nmol/L, the value of CatP4 (frozen serum) was far below what would be expected if the regression line were to be extended. This data point was considered an outlier and was removed from the regression analysis (Figure 4). Only five of 134 (3.7%) CatP4s on fresh serum (Bitch 6, times 4 and 5; Bitch 7, times 4 and 5 and Bitch 17, time 5) were above 16.2 nmol/L (Figure 1) and none of the 33 estimates of CatP4 (fresh serum) derived from the assay of frozen serum were above this cut-off.

#### Identifying the Distribution of CatP4 in the Periparturient Period

Figure 1 shows the pattern of change in CatP4 over time in 28 bitches. Visual appraisal of Figure 1 shows that the pattern of change in CatP4, as well as the levels of CatP4, generally closely parallel those of ImmP4.

**Figure 5:**
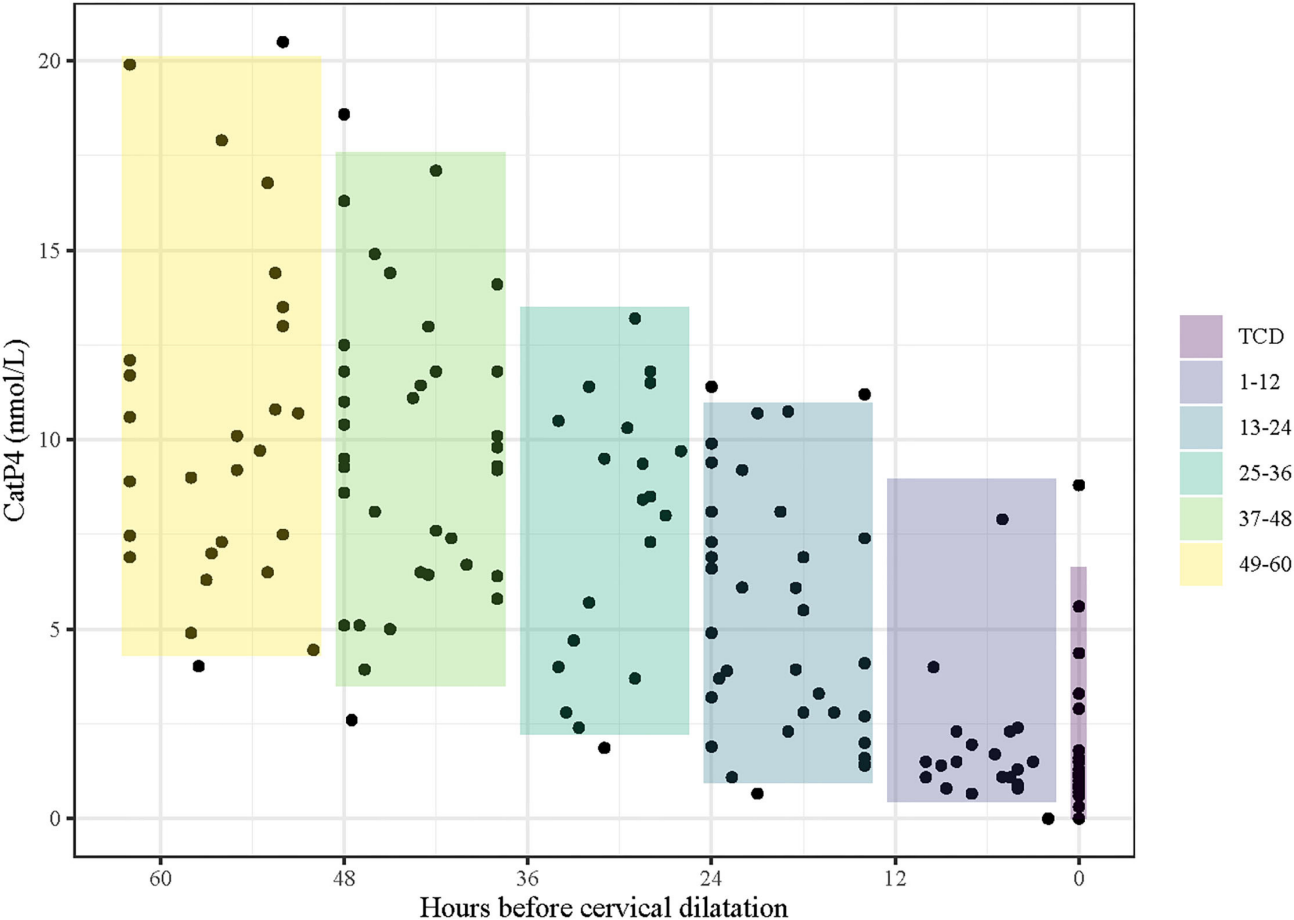
Serum progesterone levels determined with the point-of-care Catalyst assay (CatP4) at regular times every day (08h00 and 18h00) and at the time of cervical dilatation (TCD; Time 0) in 28 bitches. The data are aligned to TCD level. Shaded boxes indicate estimated distribution, determined using 100,000 bootstrapped samples and reference interval statistics to infer levels for the central 95% of a larger population similar to the study population.

Bootstrapped percentiles show expected levels of CatP4 decreasing with decreasing time to TCD (Table 4 and Figure 5). These percentiles indicate the range within which 95 percent of CatP4 levels should fall in each time interval in a population similar to the study population.

**Table 4:** Estimated lower and upper bounds for the distribution of Catalyst Progesterone concentrations (nmol/L) in whelping dogs at different time ranges before first detected cervical dilatation using bootstrapped 95-percentiles.

**H before TCD**	**Lower bound (95% CI)**	**Upper bound (95% CI)**
0	0.0 (0.0–0.7)	6.6 (3.0–8.8)
0–12	0.4 (0.0–1.0)	5.8 (2.4–7.5)
12–24	1.4 (1.3–1.9)	11.2 (9.6–11.4)
24–36	2.6 (2.4–3.8)	12.5 (11.0–13.2)
36–48	4.5 (2.6–5.7)	16.4 (14.6–17.1)
48–60	4.7 (4.7–6.4)	19.3 (13.3–20.5)
60–72	7.0 (6.9–9.2)	18.7 (10.6–19.9)

Among bitches that are <12 h prior to TCD, <2.5% are expected to have a CatP4 level >9 nmol/L (Table 4, Figure 5). Less than 2.5% of bitches that are more than 24 h prior to TCD are expected to have CatP4 levels below 2.2 nmol/L and <2.5% of those that are more than 36 h prior to TCD are expected to have CatP4 levels below 3.5 nmol/L.

## Discussion

Clinicians have limited objective tools available to assess fetal development in the dog. Ultrasonographic comparison between the fetal lung and liver has shown some promise as an indicator of fetal lung maturity, but further study is required (26). Similarly, fetal gastrointestinal motility observed *via* ultrasonography has been suggested to indicate fetal maturity (27), however, the sensitivity and specificity of this technique are too low to be clinically useful without additional confirmation (28). In the absence of clinical data from a pregnant bitch's preceding estrus (6), clinicians frequently rely on the evaluation of serum or plasma P4 levels to estimate the time to onset of spontaneous parturition. Commercially available assays for the measurement of progesterone levels use a variety of detection methods, reagents, and reference methods. These differences necessarily lead to variation in reported progesterone levels and comparisons are needed to understand these differences and support appropriate clinical interpretation. A point-of-care progesterone assay provides advantages over commercial laboratory-based assays: primarily, faster turnaround times and the increased flexibility of serial P4 monitoring. Initial point-of-care P4 assays were semi-quantitative (10) and therefore considered less accurate than RIA (29). Subsequently, a fluorescence enzyme immunoassay point-of-care assay (AIA® 360, TOSOH Corp., Minato-ku, Tokyo, Japan) has demonstrated acceptable results when compared to RIA (11), liquid chromatography/tandem mass spectrometry (30) and CLIA (31).

This study revealed that the Catalyst Progesterone performs well and has good clinical utility. The range of CatP4 evaluated in this study is relevant to bitches that are within 2½ days (60 h) of parturition, as well as periovulatory bitches sampled at the time of the LH surge and ovulation, which occur at P4 levels of around 3–6 nmol/L (1), and around 16 nmol/L, respectively (15, 32).

A negative bias was found between Catalyst and IMMULITE P4 levels that were fairly constant at levels of 7 nmol/L or lower and grew proportionally with higher levels. In contrast, Zuercher et al. (20) reported a good correlation to the CLIA but a positive proportional bias at P4 values above 6.4 nmol/L. One possible reason for this discrepancy is that Zuerchler et al. (20) assayed only frozen serum samples on the Catalyst Progesterone, rather than fresh serum as performed with the majority of samples in the current study and as recommended by the manufacturer. The outcome of a fresh vs. frozen serum comparison in the current study also showed a positive bias when frozen serum was assayed which supports this theory. Additional validation should be performed before frozen samples are recommended for routine clinical use. In the current study, bitches with >12 h to TCD had a closed cervix and a range of expected P4 values that rendered a proportional bias by the Catalyst clinically insignificant.

The results of the current study show that most bitches with 12 h or fewer to TCD will have Catalyst P4 levels below 6.6 nmol/L. Traditionally, Cesarean section in the bitch is considered safe once P4 levels drop below around 6.4 nmol/L (2ng/ml) (13). Based on the current study, this corresponds to around 4.7 nmol/L when assayed on the Catalyst.

It is worth noting that in 3 of the 28 bitches (11%) in the current study, P4 levels at the time of cervical dilatation measured ≥5.5 nmol/L on the Catalyst (ImmP4 ≥ 6.9 nmol/L). This result agrees with previous studies where 3 of 25 bitches (12%) had a P4 (measured by RIA) above 8.7 nmol/L (12) and five of 51 bitches (10%) had a P4 (measured by IMMULITE) above 6.4 nmol/L (5) at the time of cervical dilatation. Taken together, these results suggest that around 10% of bitches enter stage one of parturition with a concurrent P4 level above that traditionally considered an indication for Cesarean. In these bitches, vaginoscopy permits the timeous identification of the onset of parturition and enables Cesarean section to be performed relatively early during the whelping process. Relying solely on a drop to a specified P4 level and omitting vaginoscopy in late pregnant bitches may result in around 10% of bitches being in labor for an undefined period of time before surgical intervention is initiated, with associated risks to fetal and neonatal health and welfare.

In the current study, the CV's for CatP4 were calculated across three buckets of CatP4 levels (Table 2). The lowest bucket in which the CV for CatP4 was determined included P4 levels that are useful to identify anestrus or bitches in pro-estrus but still unlikely to have reached the LH peak (1) and the low levels found in many bitches that are within 24 h of cervical dilatation (14). The middle bucket included P4 levels associated with the LH peak or the day after the LH peak (15), as well as the levels around 8.7 nmol/L, which have been found to be a useful indicator that bitches are likely to be within 48 h of cervical dilatation (11). The high bucket (Table 2) included P4 levels associated with the time of ovulation (32) and levels around 15.8 nmol/L, which have been found useful to indicate that bitches are unlikely to be within 12 h of cervical dilatation (11). In the current study, the CVs for the Catalyst assay were generally higher than the intra- and inter-assay CVs for the IMMULITE assay (range 3.9–6.7% and 2.9–9.1%, respectively). Although the limits of the P4 buckets differ across studies, the CVs for the Catalyst assay (range 7.8–13.4%) in the current study were similar to those reported by Zuercher et al. [range 7.1–13.2%; (20)]. Previous studies in bitches have reported higher CVs for the IMMULITE than the current study, with mean intra-assay CVs ranging from 4.1 to 9.8% over a variety of P4 ranges (15, 16, 19, 33) and inter-assay CVs ranging from 6.7 to 12.9% (15, 16, 19). These CVs for the IMMULITE are comparable to the CVs for the Catalyst reported in the current study.

A limitation of the current study was the blood sampling times of 08h00 and 18h00, which were more practical for hospital staff than sampling 12-h. All P4 measurements were done on a single IMMULITE instrument and a single Catalyst instrument and therefore bias estimates may not be representative of that seen on other instruments. Serum P4 levels above 20 nmol/L were not observed in this study, so the type and amount of bias in this region cannot be determined. Furthermore, a small number of periparturent bitches (*n* = 28) was used to collect data for this study. The bootstrapping methods used in determining an expected range of P4 levels help to overcome some of the limitations of a small dataset and make the findings more applicable to the broader population of whelping bitches. However, since these bootstrapped distributions are based on the initial study population, application to dissimilar populations should be done with caution.

## Conclusion

The Catalyst Progesterone assay provides a rapid assessment of circulating P4 levels in periparturient bitches, with results comparable to those measured by IMMULITE 2000. It should be noted that while results were comparable to the IMMULITE 2000, use of the same assay for each measurement when trending progesterone levels over time is recommended to eliminate the impact of variation between different methods and improve the clinical utility of trended results.

## Data Availability Statement

The raw data supporting the conclusions of this article will be made available by the authors, without undue reservation.

## Ethics Statement

The animal study was reviewed and approved by Animal Ethics Committee, University of Pretoria, South Africa. Written informed consent was obtained from the owners for the participation of their animals in this study.

## Author Contributions

KD: conceptualization, investigation, project administration, and writing the original draft. JN: conceptualization, methodology, supervision, formal analysis, and writing the original draft. CJ: writing the original draft and writing, reviewing, and editing the manuscript. ES: conceptualization and writing, reviewing, and editing the manuscript. EH: statistical analysis and writing, reviewing, and editing the manuscript. All authors contributed to the article and approved the submitted version.

## Conflict of Interest

ES and EH are full time employees of IDEXX Laboratories, Inc. KD has acted as a consultant and speaker for IDEXX Laboratories, Inc. The remaining authors declare that the research was conducted in the absence of any commercial or financial relationships that could be construed as a potential conflict of interest.

## Publisher's Note

All claims expressed in this article are solely those of the authors and do not necessarily represent those of their affiliated organizations, or those of the publisher, the editors and the reviewers. Any product that may be evaluated in this article, or claim that may be made by its manufacturer, is not guaranteed or endorsed by the publisher.
